# Methadone Patient Access to Collaborative Treatment: Protocol for a Pilot and a Randomized Controlled Trial to Establish Feasibility of Adoption and Impact on Methadone Treatment Delivery and Patient Outcomes

**DOI:** 10.2196/69829

**Published:** 2025-04-15

**Authors:** Beth E Meyerson, Alissa Davis, Richard A Crosby, Linnea B Linde-Krieger, Benjamin R Brady, Gregory A Carter, Arlene N Mahoney, David Frank, Janet Rothers, Zhanette Coffee, Elana Deuble, Jonathon Ebert, Mary F Jablonsky, Marlena Juarez, Barbara Lee, Heather M Lorenz, Michael D Pava, Kristen Tinsely, Sana Yousaf

**Affiliations:** 1 Harm Reduction Research Lab University of Arizona College of Medicine-Tucson Tucson, AZ United States; 2 Comprehensive Center for Pain and Addiction University of Arizona Health Sciences, University of Arizona Tucson, AZ United States; 3 School of Social Work Columbia University New York, NY United States; 4 College of Public Health University of Kentucky Lexington, KY United States; 5 College of Health and Human Services School of Interdisciplinary Health Programs Western Michigan University Kalamazoo, MI United States; 6 School of Nursing Indiana University Bloomington, IN United States; 7 Southwest Recovery Alliance Phoenix, AZ United States; 8 School of Global Health New York University New York, NY United States; 9 StatLab BIO5 Institute University of Arizona Tucson, AZ United States; 10 College of Nursing University of Arizona Tucson, AZ United States; 11 Community Medical Services Phoenix, AZ United States; 12 Drug Policy Research and Advocacy Board Tucson, AZ United States

**Keywords:** methadone, implementation, patient-centered treatment, opioid use disorder, posttraumatic stress symptoms, vicarious trauma

## Abstract

**Background:**

Access to methadone treatment can reduce opioid overdose death by up to 60%, but US patient outcomes are suboptimal. Federally allowed methadone treatment accommodations during the COVID-19 public health emergency were not widely adopted. It is likely that staff-level characteristics such as trauma symptoms influence the adoption of treatment innovation.

**Objective:**

Methadone Patient Access to Collaborative Treatment (MPACT) is a 2-phased project (pilot and field trial) to develop and test a staff-level, multimodal intervention to increase staff adoption of low-barrier, patient-centered methadone treatment practices and ultimately improve treatment retention and patient outcomes.

**Methods:**

A pilot and national trial will measure implementation feasibility, acceptability, and effects of the MPACT intervention on treatment practice change, clinic culture, patient retention, and patient posttraumatic stress symptoms (PTSS). The pilot will be a single-arm 5.5-month pilot study of MPACT conducted in 2 Arizona methadone treatment clinics (rural and urban) among 100 patients and 22 staff. The national trial will be a 20-month cluster randomized trial conducted among 30 clinics, 600 patients (20 per clinic), and 480 staff (18 per clinic). Data will be gathered by staff and patient surveys and patient chart review. The primary study outcome is increased patient methadone treatment retention measured as (1) time to first treatment interruption from study enrollment; (2) active in treatment at enrollment, day 30, 60, 90, and 120; and (3) continuous days in treatment during the study period. Secondary study outcomes include reductions in vicarious trauma and PTSS among enrolled opioid treatment program staff and PTSS among enrolled patients.

**Results:**

The pilot study was funded by the National Institute on Drug Abuse (award R61DA059889, funded September 2023), and the field trial will be funded under the associated R33 mechanism in September 2025. The pilot study was completed in March 2025. The randomized controlled trial will begin in December 2025. Both the pilot and trial have been approved by the University of Arizona Human Subjects Protection Program and have been registered with the clinical trials network.

**Conclusions:**

The MPACT study will provide a foundation for an evidence-based, staff-level intervention aimed at improving patient retention in methadone treatment. Future studies should examine the individual components of MPACT to determine their differential contributions to the primary outcome of patient methadone treatment retention and to secondary outcomes of staff and patient reduction in stress symptoms.

**Trial Registration:**

ClinicalTrials.gov NCT06513728; https://clinicaltrials.gov/study/NCT06513728 and ClinicalTrials.gov NCT06556602; https://clinicaltrials.gov/study/NCT06556602

**International Registered Report Identifier (IRRID):**

DERR1-10.2196/69829

## Introduction

### Background

Methadone is one of the most essential tools available to reduce opioid overdose deaths because it is safe, effective, and patient-preferred for the treatment of opioid use disorder (OUD) [1-3]. Access to methadone, one of 2 safe and effective OUD treatments, can reduce overdose mortality by up to 60% [4,5]. However, the promise of methadone is significantly diminished by geographic maldistribution of clinics and variations in the delivery of methadone maintenance treatment (MMT) across the country [6-8]. Treatment variations likely produce the observed wide-ranging MMT retention rates (30%-84%) [9,10].

MMT in the United States is delivered only by opioid treatment programs (OTPs; “methadone clinics”) certified and accredited by the federal government [11]. Variation in treatment quality and access maldistribution means that the impact of poor MMT outcomes is felt most acutely in rural communities as well as among populations who are Black, Hispanic, or Indigenous [12]. Unlike other health care environments, OTPs serve a daily average of more than 100 people in a narrow time window [13] and have been described as feeling “like bus stations” [14] rather than medical clinics. OTPs have been criticized as being unresponsive to patient need for treatment flexibility [15] and are not equipped to address what we know to be higher rates of patient trauma exposure and posttraumatic stress symptoms (PTSS) compared to the general population [16,17]. While MMT outcomes can be impeded by patient trauma [18], it is also possible that poor MMT outcomes and patient trauma are exacerbated by OTP practice and culture [19,20]. Patients report disenfranchisement from treatment decision-making through language referring to dosing as “privileges,” staff behavior described as “carceral” [21], and being tied to the OTP by “liquid handcuffs” due to daily required in-clinic supervised dosing [22].

**Figure 1:**
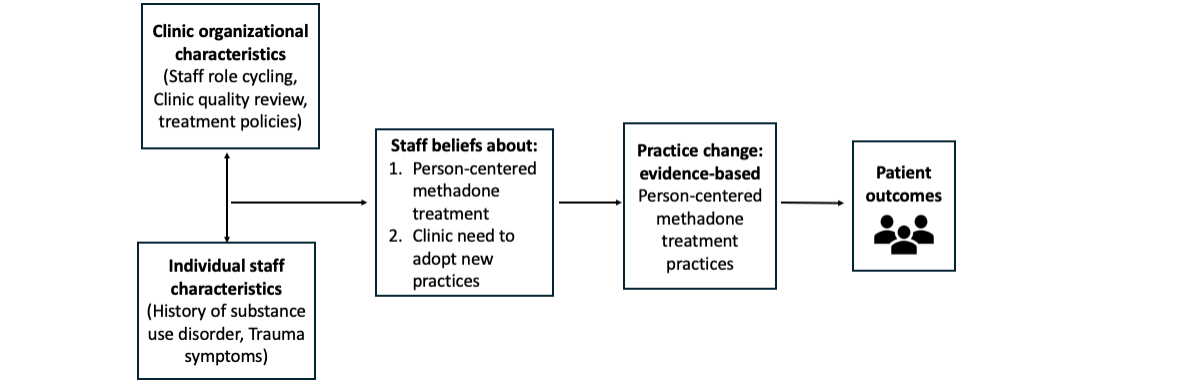
Potential inner setting factors impacting methadone treatment outcomes.

Policy and systems evolution is occurring to improve the way MMT is delivered in the United States. Unprecedented US regulatory change during the COVID-19 public health emergency [23] and again in February 2024 [11] permitted and then further clarified methadone dosing and delivery flexibility so that treatment was more individualized and patient-centered. However, as has been observed, policy changes during the COVID-19 public health emergency were insufficient to ensure sustained changes [24-26]. This is likely the result of multiple factors hindering the implementation of MMT innovation. Implementation science suggests that in addition to the outer setting factor of federal policy, there are inner setting factors that likely influence the adoption of MMT treatment innovation [27]. These include clinic organizational characteristics and culture as well as staff characteristics and staff beliefs. Figure 1 displays our current thinking about hypothesized relationships between and among inner setting factors, adoption of innovation, and patient outcomes.

Staff trauma is one particular inner setting factor that is linked to the adoption of innovation and quality of treatment delivery. A preliminary study by several authors here suggests that OTP staff trauma may play a central role in shaping clinic culture and methadone treatment practice changes [14]. Evidence from studies among other types of health professionals demonstrates that vicarious trauma (VT), or work-related trauma (ie, coexperiencing patient distress and change in worldviews because of ongoing distress), is associated with reduced staff empathy and increased PTSS [28]. VT outcomes include burnout, reduced patient empathy and compassion satisfaction, low morale, impaired clinical decision-making, and compromised patient care [29-31]. The only extant study of OTP staff trauma histories and symptoms found that 63% of staff exhibited PTSS at clinical levels, indicating a need for treatment [32]. Therefore, a potential strategy to facilitate the adoption of MMT innovation is to implement staff-level interventions aimed at reducing PTSS and VT while providing training about low-barrier, patient-centered methadone treatment.

To this end, we developed Methadone Patient Access to Collaborative Treatment (MPACT): a multimodal intervention to increase staff awareness of and readiness to adopt MMT treatment innovation. MPACT promotes treatment flexibilities allowed by federal regulators and patient-centered, trauma-informed MMT and seeks to empower OTP staff and clinic groups to adopt these treatment flexibilities by addressing staff VT and PTSS, which will improve treatment quality and ultimately MMT retention. The objective of this study is to test the adoption, feasibility, and impact of MPACT on methadone treatment delivery and patient outcomes. There are 6 specific aims for the MPACT study over the 6-year project period. The specific aims are listed here and will be described in the following subsections.

### Phase 1, Years 1-2: MPACT Intervention Development and Pilot Testing

Phase 1 aims (1) to develop MPACT through multilevel, iterative planning with methadone clinic staff and people with recent methadone treatment experience; (2) to determine MPACT implementation feasibility, acceptability, and preliminary effect on methadone treatment practice change and clinic culture; and (3) to determine the preliminary effect of MPACT on methadone treatment retention and patient PTSS.

### Phase 2, Years 3-6: Hybrid, Cluster Randomized Controlled Trial

Phase 2 aims (4) to quantify the effects of MPACT on methadone treatment practice change and clinic culture, (5) to determine the efficacy of MPACT on methadone treatment retention and patient and staff PTSS outcomes, and (6) to evaluate the effect of patient and staff trauma on primary outcomes and staff MPACT implementation.

## Methods

### Ethical Considerations

The MPACT study protocol and related documents were reviewed and approved by the University of Arizona Human Subjects Protection Program (pilot: #STUDY00003631 and trial: #STUDY00005677), the single institutional review board overseeing all sites participating in the study: University of Arizona, Indiana University, Western Michigan University, and Columbia University. All participants will engage in a web-based informed consent process prior to study enrollment. The consent will be downloaded and retained by the study as documentation. Patient participants will consent to both survey participation and the release of specified elements of their clinic medical record for the purpose of the study. Confidentiality of staff and patient participants in the enrolled MPACT clinics will be preserved by making every effort to prevent the clinic leadership and staff from knowing which patients are enrolled as study participants and keeping clinic leadership and patients from knowing which staff are enrolled as study participants. Unique identifiers will be created at the time of enrollment and used throughout the study period. All study personnel (staff and investigators) have been trained in human participant protection through the completion of Social Behavioral Research and Biomedical Research modules with the Collaborative Institutional Training Initiative program and the completion of conflict of interest training and have annually declared conflicts of interest for review by the University of Arizona Human Subjects Protection Program. All reported data will be aggregated and deidentified. All information will be stored in a secure and encrypted drive and accessible only by the principal investigator (BEM) and the study coordinator (SY). The study was registered under ClinicalTrials.gov (NCT06513728 for the phase 1 pilot and NCT06556602 for the phase 2 trial). Participants will be offered financial remuneration totaling US $100 for the completion of all 5 surveys on time and during the pilot study period and US $160 for the completion of surveys on time and during the trial study period.

### Phase 1, Years 1-2: MPACT Intervention Development and Pilot Testing

#### Overview

- Aim 1: Develop MPACT through multilevel, iterative planning with methadone clinic staff and people with recent methadone treatment experience.

MPACT is an experimental intervention comprised of 4 evidence-based components adapted by a group of people who have been in methadone treatment within the past 5 years in Arizona, a group of OTP staff in all clinic roles (front desk, peer support staff, case management, counseling, clinical supervision, medical, and administrative) from 3 Arizona OTPs (2 urban and 1 rural), and a group of subject matter experts focused on clinical supervision, human resources, and employee education.

**Figure 2:**
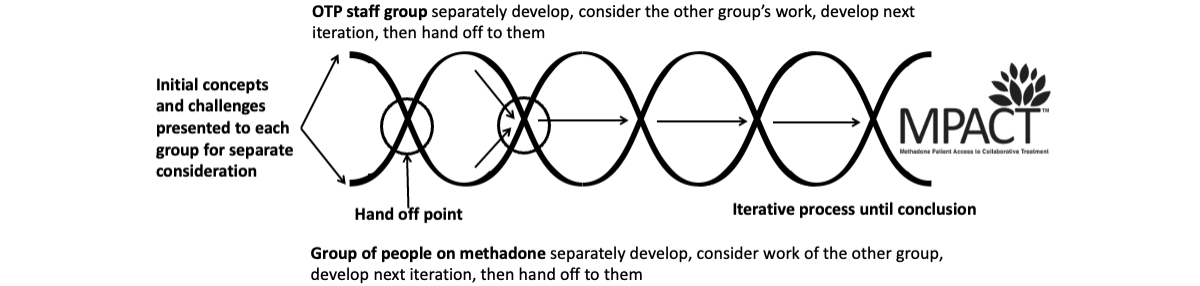
Trauma-informed, collaborative development structure to refine MPACT components. MPACT: Methadone Patient Access to Collaborative Treatment; OTP: opioid treatment program.

The adaptation of MPACT components was accomplished through an iterative codevelopment process involving OTP staff and methadone community (patient) groups. The creation of a trauma-informed codevelopment space was crucial to facilitate safer and more open discussions. To accomplish this, we established a parallel, intervention refinement process using a helical structure developed by this team and based on our prior research with structural indicators for community-based participatory action research [33]. As shown in Figure 2, the “hand off” of work drives an iterative (helical) thinking process. This structure provides distinct spaces for thoughtful dialogue within and between each group.

The outcome of the codevelopment process was a robust multimodal intervention (MPACT) comprised of the following 4 elements.

#### Accredited Psychoeducational Training

A jointly accredited, self-paced, 3-module psychoeducational training focused on (1) the definition and application of low-barrier, patient-centered, trauma-informed methadone treatment; (2) public and clinic policy (federal and state); and (3) clinic staff opportunities to increase patient-centered, trauma-informed methadone treatment. The training seeks to empower staff to initiate any positive change at the individual and staff group levels. Joint accreditation offers continuing medical education for physicians and nurses as well as continuing education credits for social workers, psychologists, peer support specialists, case managers, and administrators. Training completion is incentivized by the award of 3 free continuing education credits according to professional discipline. While training is voluntary, to receive the continuing education credits, staff of MPACT-enrolled clinics must complete the training within 2 weeks of the MPACT launch within the clinic. New staff can complete the training as they are hired during the MPACT intervention period. This modular training approach was adapted from a prior successful project focused on increasing pharmacy syringe sales to people who use drugs [34].

#### Staff Wellness Education and Assessment

All staff in MPACT-enrolled clinics will receive training about trauma exposure and reactions, trauma-informed methadone treatment, availability and modalities of trauma treatment, and VT through curated presentation materials. These materials will be accessible to all staff through an MPACT web portal and through training or communications as determined by the enrolled clinics. The training materials include video and visual content designed for easy integration into clinic employee training, onboarding, or as refresher training. As part of the training, staff will be introduced to an anonymous web-based “wellness” screener, which includes an 8-item posttraumatic stress disorder symptoms screener (Posttraumatic Stress Disorder Checklist [PCL-5]) [35,36] and an 8-item Vicarious Trauma Scale [37]. Individual screening outcomes (results) trigger a curated and immediately presented message regarding self-care, referral to the VA PTSD Coach [38,39] (a downloadable, free application) and referral to the employee assistance program offered as a behavioral health benefit to employees where indicated. Employees will have access to wellness training throughout the MPACT intervention period and can use the self-screener repeatedly if desired. The screener will also be “advertised” in staff-only areas with a curated poster on stress, including a link or QR for easy access.

#### Trauma-Informed Clinic Self-Assessment

A trauma-informed clinic self-assessment (TICA) will be conducted quarterly during the study period. TICA assessment outcomes are generated by data from aggregated responses to a 16-item anonymous survey of staff measuring staff development, available resources, support, safe physical environments, trauma-informed policies, and patient-centered practices specific to methadone clinics. Items were selected and modified by the study team using an organizational trauma-informed practice tool as a reference instrument [40]. Summarized results are discussed with clinic leadership who will decide how and when to share them with clinic staff.

#### Reflective Supervision Consultation

Reflective supervision is an evidence-based professional development intervention focusing on the relationship and process of collaborative case consultation and reflection for clinicians providing psychosocial support [41,42]. Reflective supervision provides strategic guidance to increase self-reflectiveness and self-awareness and encourages participants to independently process clinical encounters and solve challenges. These skills have been shown to improve patient care [43,44]. To our knowledge, there are no existing reflective supervision consultation models tailored to OTP staff. Therefore, we adapted the standard reflective supervision practices to apply and be accessible to all OTP staff who have intensive and consultative interactions with patients. These staff roles include case managers, counselors, and peer support. Reflective supervision will begin in month 1 of the intervention period and will continue on a biweekly basis throughout the intervention period for each MPACT-enrolled clinic. Sessions are facilitated by a trained reflective supervisor who will also be trained by the MPACT study clinician.

- Aim 2: Determine MPACT implementation feasibility, acceptability, and preliminary effect on methadone treatment practice change and clinic culture.
- Aim 3: Determine the preliminary effect of MPACT on methadone treatment retention and patient PTSS.

A single-arm 5.5-month pilot study of MPACT will address aims 2 and 3 and involves 2 Arizona-based OTPs (1 rural and 1 urban), 100 patients, and 22 staff (25 patients and 6 staff of the rural clinic and 75 patients and 16 staff of the urban clinic). Data collection will be accomplished through a web-based survey of staff and patient participants monthly during the pilot study period, which began in October 2024 and ended in March 2025. The 4 elements of MPACT intervention were delivered during the 4-month period following study recruitment. Eligibility criteria for study inclusion included being 18 years of age or older, being a staff member or a patient at 1 of the 2 pilot clinics, being willing to participate in monthly surveys during the pilot study period, and (for patients) agreeing to share selected components of their medical charts with the study team.

### Measures

The primary study outcome is increased patient methadone treatment retention. This outcome is measured in three ways: (1) time to first treatment interruption, calculated as the number of days to first missed dose from day 0 (MPACT enrollment); (2) evidence of being active in treatment, a binary (yes or no) if receiving dose at points in time on day 0, 30, 60, 90, and 120; and (3) continuous days in treatment during the study period, calculated as time (days) to discharge. Data measuring this outcome are gathered by patient surveys and chart reviews.

Secondary study outcomes include reductions in VT and PTSS among enrolled clinic staff and PTSS among enrolled patients. Data measuring secondary outcomes are gathered by a survey of staff and patients enrolled in the study. For staff and patients, PTSS are measured using the 8-item posttraumatic stress disorder symptoms screener otherwise known as the PCL-5 [35]. Staff VT is measured by the Vicarious Trauma Scale [37], burnout is measured by a 3-item scale [45], and compassion satisfaction and compassion fatigue are measured by the shortened, 9-item Professional Quality of Life Scale for staff [46].

The degree to which methadone treatment is patient-centered is also a secondary outcome measured through staff surveys (assessing whether they believe they are providing it) and patient surveys (assessing whether they feel they are experiencing it). Patient-centered care competency is measured by a 19-item scale [47] including the following subscales: respecting patient perspectives, promoting patient involvement in the care process, providing patient support, and advocating for patients. Patient-centered care, as defined by the study team, is measured using a 5-item instrument that reflects the concepts of patient-centered care introduced during the accredited training modules. This scale is administered to staff, with an adapted version used for patients.

Other individual-level variables of interest for the staff participants include (1) personal characteristics—demographics, personal substance use disorder and treatment experience, and trauma exposure history (measured by the Life Events Checklist-5) [48]; (2) work characteristics—training, education, and licensure related to their clinic role; (3) empowerment using a 5-item empowerment scale [49]; (4) stigma—toward people with OUD with a 9-item scale [50], self-stigma with a 9-item scale [51], and fear of enacted stigma through a 9-item scale [52]; (5) beliefs—about trauma-informed care measured by the attitudes toward trauma-informed care [53] and about abstinence measured by the Abstinence Orientation Scale [54]; (6) comfort with targeted practices related to the most recent federal changes to methadone treatment delivery measured by items adapted from prior studies measuring comfort with practices [34,55,56]; and (7) fidelity to MPACT—the degree to which the clinic implements the MPACT intervention.

Other individual-level variables of interest for the patient participants include (1) personal characteristics—demographics, housing, trauma history (Life Events Checklist-5), and trauma symptoms (PCL-5); (2) methadone treatment—time in treatment and dose sufficiency; (3) empowerment—as measured by a 15-item scale [57] and through an adapted 16-item Kim Alliance Scale [58]; and (4) fidelity to MPACT—the degree to which the clinic implements the MPACT intervention.

As this is a hybrid (implementation and effectiveness) pilot and trial, we are specifically focused on reach, implementation, adoption, and (in the trial) maintenance using the RE-AIM (Reach, Effectiveness, Adoption, Implementation, and Maintenance) framework [59]. Measures collected for the pilot will also be collected for the trial.

### Data Collection

Primary and secondary study outcomes will be measured by surveys and patient chart reviews. Surveys will be administered monthly for the pilot study: assessment 1 (baseline at enrollment) and assessment 2-5 in 30-day sequences through the study period, with a contact reminder at day 27 and a completion forgiveness period of 5 days (day 35). Table 1 displays the sequencing of measures across the 5 pilot surveys.

**Table 1:** Sequencing of Methadone Patient Access to Collaborative Treatment (MPACT) primary and secondary measures (assessment 1-5).

Construct		Assessment 1 (enrollment)			Assessment 2		Assessment 3		Assessment 4		Assessment 5 (study conclusion)
		Staff items, n	Patient items, n								
**Personal characteristics**											
	Demographics	5	6	—^a^		—		—		—	
	Personal SUD^b^ experience	10	—	—		—		—		—	
**Methadone treatment**											
	Time in MMT^c^ and clinic, reasons for choosing methadone as treatment	—	13	—		—		—		—	
	Methadone interruption	—	2	✓		✓		✓		✓	
	Dose, sufficiency, OD^d^	—	13	✓		✓		✓		✓	
**Trauma history and symptoms**											
	Trauma history (LEC^e^)	17	17	—		—		—		—	
	Vicarious Trauma Scale	8	—			✓				✓	
	PCL-5^f^ (trauma symptoms)	8	8	✓^g^		✓		✓^g^		✓	
**Burnout, compassion fatigue**											
	Burnout scale	9	—	✓		✓		✓		✓	
	ProQoL^h^ (compassion fatigue, Compass Sat, burnout)	9	—	✓		✓		✓		✓	
**Work characteristics**											
	Years working (SUD and this clinic) and role	5	—	—		—		—		—	
	Training and education for role	2	—	—		—		—		—	
**Baseline exposure to MPACT-related practices**											
	Reflect Sup (some staff)	1	—	✓		✓		✓		✓	
	Self-care	9	—	—		✓		—		✓	
	MPACT-specific practices	9	—	✓		✓		✓		✓	
**Empowerment**											
	Staff empowerment scale	5	—	—		✓		—		✓	
	Patient empowerment (Bann scale)	—	15	✓		✓		✓		✓	
**Beliefs**											
	Abstinence Orientation Scale	11	—	—		✓		—		✓	
	Comfort with MMT innovations	10	—	—		✓		—		✓	
	ARTIC^i^	10	—	—		✓		—		✓	
	Person-centered climate (PCQ-S^j^)	5	—	—		✓		—		✓	
**Stigma**											
	Stigma toward people with OUD^k^	8	—	—		✓		—		✓	
	Self-stigma	9	—	—		✓		—		✓	
	Fear of enacted stigma	9	—	—		✓		—		✓	
**Patient-centered care practices**											
	PCC^l^	19	—	—		✓		—		✓	
	Team-derived PCC scale	6	6	✓		✓		✓		✓	
	Kim Alliance Scale	—	16	✓		✓		✓		✓	
**Implementation**											
	MPACT feasibility, accept and fit; likelihood of continuing practices	5	—	—		—		—		✓	

^a^Not applicable.

^b^SUD: substance use disorder.

^c^MMT: methadone maintenance treatment.

^d^OD: opioid overdose.

^e^LEC: Life Events Checklist.

^f^PCL-5: Posttraumatic Stress Disorder Checklist.

^g^Patients only.

^h^ProQoL: Professional Quality of Life Scale.

^i^ARTIC: attitudes toward trauma-informed care.

^j^PCQ-S: Patient-Centered Climate Scale for staff.

^k^OUD: opioid use disorder.

^l^PCC: person-centered competence.

For the 20-month trial, there will be 8 surveys from baseline assessment at enrollment through the remaining 7 assessments conducted every 77 days. Survey responses will be collected using the Qualtrics platform, accessible directly by participants.

For all enrolled patients, a review of their methadone clinic medical chart will include the duration of their treatment at the clinic, from treatment initiation to discharge or study end, whichever occurs first. This review will take place at the conclusion of the study period in accordance with the data sharing agreement (DSA) established between the clinic organization and the University of Arizona. A feasibility test with a sample of 50 charts with deidentified data was conducted in June 2024 and confirmed timely data transfer, data completeness, and utility for outcome measurement for the pilot clinics. Our national survey of OTP clinic directors found that 22.2% of clinics allowed DSAs with researchers, but the vast majority (77.3%) indicated that such agreements were not allowed or that they were not aware of the clinic organization’s position on them [60]. For the purposes of the trial, then, only clinics allowing DSAs will be eligible for study enrollment. The following medical chart segments will be requested for each patient participant: (1) the digest of the patient history of starting and leaving treatment at that clinic (dates), (2) case notes, (3) discharge summary, (4) treatment plans, (5) milligram dosing, and (6) take-home medication status over time. Case notes include qualitative data on patient stability, challenges reported by the patient (eg, housing, transportation, safety, and dosing sufficiency), and instances of missed doses. For the trial, the data will be transferred using unique identifiers that will correspond with the study unique identifiers. No personally identifying information (name and street address) will be transferred.

### Study Recruitment

Recruitment is stepwise for both the pilot and the trial. For the national trial, clinics will first be recruited through email from a national list of methadone clinics responding to a prior survey by this team during 2024 [60]. A second strategy will involve an email to the state opioid treatment authority with a request to forward study information and the recruitment flyer to methadone clinic directors in their state. State opioid treatment authorities are the single opioid regulator in each state. Clinics that allow study recruitment among staff and patients, establish a DSA for the transfer of patient participant methadone treatment chart data at the conclusion of the 20-month trial, and identify a clinic “champion” to assist with study enrollment and study contact will be eligible for randomization as described below.

Following clinic enrollment, each clinic champion will post recruitment flyers in staff-only areas (for staff participants) and in patient-only areas (for patient participants). Recruitment flyers for staff lead to a study portal (web-based) presenting information about the study and requesting agreement to participate. If agreement is made, staff participants will immediately complete the enrollment survey (baseline assessment 1). The same process will occur for patients. Payment for timely completion of each survey is US $20 for patients and staff. This cost was determined by a group of staff and patients who completed the work associated with aim 1.

Patient and staff confidentiality will be maintained by centralizing the enrollment process. Recruitment flyers will be displayed in staff-only and patient-only areas with a QR code or URL leading to information for potential participants to learn more about the study and to voluntarily enroll. This process ensures the anonymity of study participants within the clinic, meaning that patients and staff participants will not be known to the study clinic. Further, at the time of enrollment, a unique identifier will be established by the participant and will be used henceforth. At no time will the clinic leadership or clinic champion know the identity of the study participants. The only exception to this is at the conclusion of the study when patient chart data transfer will occur, and at that time, only 1 person handling data transfer will have the name and dates of birth of the participants whose charts will be transferred for study purposes.

### Fidelity Tracking and Application of RE-AIM

MPACT fidelity tracking will assess the degree to which clinics assigned to the intervention arm implement MPACT components. This will be evaluated by a fidelity tracking instrument and video conversations with the clinic champions—biweekly during the pilot study and monthly during the trial period. A fidelity tracker will first be populated with data from study databases, including accredited training completion, TICA survey participation (number and ratio of staff completing surveys), number of anonymous wellness self-screenings, and reflective supervision participation (number of staff by role per biweekly session). During fidelity conversations, the clinic champion will indicate the number of wellness trainings offered to staff (current or new staff) in the past 2 weeks, whether posters for the wellness screener were shared in the staff-only areas, and whether there were other issues raised by staff about MPACT participation that may require troubleshooting.

MPACT feasibility will be measured through fidelity tracking (what the clinic does and does not implement and the feedback about issues related to that) and through items measured in the staff survey. The RE-AIM framework will guide the assessment of reach, adoption, implementation, effectiveness, and maintenance. Reach is focused on staff and patient participants in the MPACT study. Measures of reach include the number and proportion of clinics participating in the study (number participating/number in recruitment sample) and the number and proportion of staff and patients participating by the study clinic. Adoption is focused on the participation in MPACT intervention overall and by the MPACT component. This is measured by reported staff participation through the surveys and through completion data gathered through the intervention components. Examples include accredited training completion or progress toward completion, reflective supervision participant reports, and wellness assessment completion reported through study surveys and through wellness screening data output (duplicated unless noted by the participant by selecting “I have taken this assessment before,” and if selected, the participant can select the number of times the assessment has been completed prior). Implementation is measured through the fidelity check meetings with champions and reported MPACT activity (such as posting flyers about particular intervention components). Effectiveness is measured by primary and secondary outcomes related to the aims of this study. Maintenance will not be measured in the pilot study (4-month period) but will be measured through a survey conducted 6 months after the conclusion of the trial period.

### Phase 2, Years 3-6: Hybrid, Cluster Randomized Controlled Trial

#### Overview

- Aim 4: Quantify the effects of MPACT on methadone treatment practice change and clinic culture.
- Aim 5: Determine the efficacy of MPACT on methadone treatment retention and patient and staff PTSS outcomes.
- Aim 6: Evaluate the effect of patient and staff trauma on primary outcomes and staff MPACT implementation.

Findings from the pilot study will determine the preliminary effect size to confirm power analyses and final sampling for a hybrid type 1, 20-month cluster randomized controlled trial among 30 clinics, 600 patients (20 per clinic), and 480 staff (18 per clinic). This hybrid type 1 trial will focus primarily on MPACT’s effect outcomes while examining the association of MPACT implementation fidelity and acceptability and identifying the multilevel factors influencing implementation.

For the trial, the clinic is the unit of randomization. The intervention condition will be the MPACT intervention, and the control condition will involve accredited training about methadone that does not overlap aspects of the MPACT intervention. As shown in Table 2, we will allow a 20-month study period to accommodate staggered trial initiation through month 12 of year 2. Given the 20-month trial period, we will allow for new staff members to enroll through the end of the 7th month of their site’s trial period.

**Table 2:** Cluster randomized controlled trial of Methadone Patient Access to Collaborative Treatment.

	Year 1			Year 2			Year 3			Year 4	
	Half 1	Half 2	Half 1		Half 2	Half 1		Half 2	Half 1		Half 2
Intervention arm clinics (n=15)	Start-up	Intervention	Intervention		Intervention	Intervention		Intervention	Intervention		Closeout
Control arm clinics (n=15)	Start-up	Usual care	Usual care		Usual care	Usual care		Usual care	Usual care		Closeout
Key processes	Randomization process finalized	Trial allows for staggered starts based on recruitment	Trial allows for staggered starts based on recruitment		Trial allows for staggered starts based on recruitment	Trial follow-up		Trial follow-up	Trial follow-up		Trial outcome analyses and dissemination

#### Clinic Stratification Factors

By the time of trial planning finalization, we anticipate that the state regulatory environment in each trial location will be a likely outer setting impact. Given the importance of state policy for regulating OTPs and methadone treatment, we will measure state regulatory favorability toward OTPs using a 2-level coding structure used in prior studies by this team [60]. We will code state laws based on the Pew state regulatory review [8] as “expanding methadone access” or “not expanding or limiting access.” Randomization to trial condition will be stratified based on outcomes of this state regulatory coding.

### Statistical Analysis

Our primary outcome is patient time to first treatment interruption (confirmed in the pilot). Secondary outcomes include treatment retention (yes or no) at selected time points (1, 3, 6, and 12 months) and time to treatment discontinuation. To accommodate the clustering induced by nesting patients within clinics, we will use a mixed effects Cox proportional hazards model (shared frailty model) [61,62] to accommodate differential survival probability among clusters. The mixed model will include a random intercept for the clinic and a fixed treatment effect for MPACT or control assignment. We will also include patient-level covariates for age, sex, and time in MMT.

**Figure 3:**
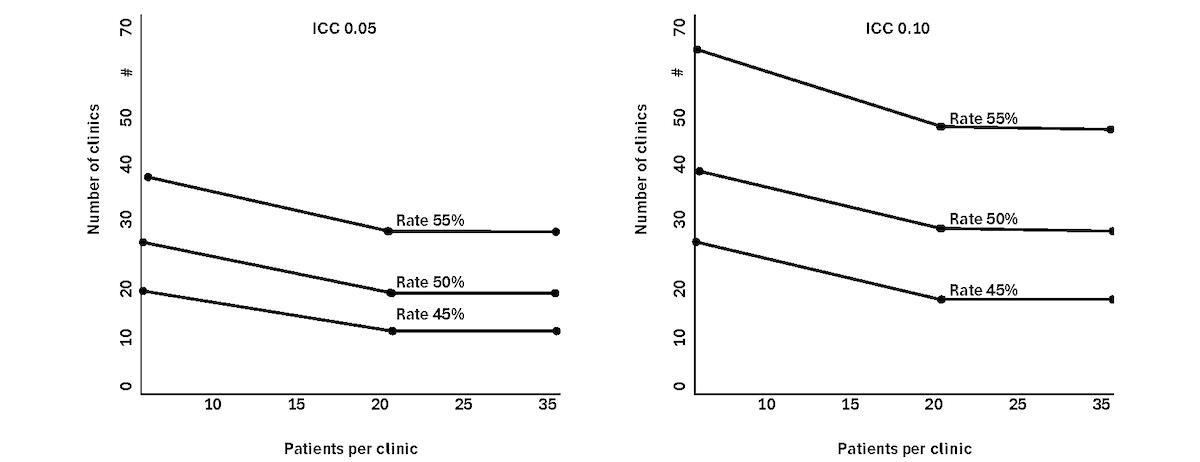
Number of clinics and patients for 80% power (vs 66% treatment interruption rate), MPACT trial. ICC: intraclass correlation coefficient; MPACT: Methadone Patient Access to Collaborative Treatment.

Our initial sample size calculation uses asymptotic normal results for log hazard ratio as well as sample size inflation factors (eg, Donner) [62] for cluster randomized trials. In designing the future R33 trial, we will make use of specific sample size methods for cluster randomized trials with time-to-event outcomes [63,64]. The relative frequency of first treatment interruption [65] is estimated as 66% at 12 months of MMT until we have confirmation from the pilot. We evaluate the number of clinics and number of patients, assuming that MPACT intervention reduces this frequency to 45% (n=240), 50% (n=300), and 55% (n=330). The power curves based on independent observations (no cluster effect) are shown in Figure 3. The graph shows that the recruitment of 30 clinics, with 20 patients per clinic, provides greater than 80% power to detect a difference in treatment interruption rates of 66% (control) and 55% (MPACT) with α=.05. Consistent with the cluster randomization trial design, we also consider the average number of patients per clinic as 10, 20, and 40 and different degrees of intraclinic clustering using intraclass correlation coefficients of 0.05 and 0.10.

## Results

The pilot study is funded by the National Institute on Drug Abuse (award R61DA059889, funded September 2023), and the field trial will be funded under the associated R33 mechanism in September 2025. The pilot study was completed on March 17, 2025. We are currently analyzing the pilot study findings. The randomized controlled trial will begin in December 2025.

## Discussion

The MPACT study will provide a foundation for an evidence-based, staff-level intervention aimed at improving patient retention in MMT. We anticipate a decrease in reported levels of VT and PTSS among staff and an increase in methadone treatment retention among patients. The pilot outcomes are focused primarily on implementation with a preliminary indication of impact or effectiveness. The preliminary outcomes from the pilot will inform the final sampling to properly power the study. The trial outcomes are focused both on the implementation and effectiveness of the MPACT intervention. Future studies should examine the individual components of MPACT to determine their differential contributions to the primary outcome of patient MMT retention and to secondary outcomes of staff and patient reduction in stress symptoms.

## Appendix

Multimedia Appendix 1 Peer review report from ZDA1 SKP-E (O2) - National Institute on Drug Abuse Special Emphasis Panel HEAL Initiative: Translating Research to Practice to end the Overdose Crisis (National Institutes of Health, USA).

## Abbreviations

DSA: data sharing agreement
MMT: methadone maintenance treatment
MPACT: Methadone Patient Access to Collaborative Treatment
OTP: opioid treatment program
OUD: opioid use disorder
PCL-5: Posttraumatic Stress Disorder Checklist
PTSS: posttraumatic stress symptoms
RE-AIM: Reach, Effectiveness, Adoption, Implementation, and Maintenance
TICA: trauma-informed clinic self-assessment
VT: vicarious trauma

## Data Availability

The datasets generated and analyzed during this study will be made available in the HEAL data ecosystem through the Inter-University Consortium for Political and Social Research within 1 year of the pilot and of the trial conclusion, or at the time of first publication from the pilot (or trial), whichever occurs first.
