# Supplementary material for: Methadone Patient Access to Collaborative Treatment: Protocol for a Pilot and a Randomized Controlled Trial to Establish Feasibility of Adoption and Impact on Methadone Treatment Delivery and Patient Outcomes
Source: JMIR Res Protoc. 2025 Apr 15;14:e69829. doi: 10.2196/69829 (PMC12041824; doi:10.2196/69829)
Supplement: Multimedia Appendix 1 [file resprot_v14i1e69829_app1.pdf]

1R61DA059889-01 Meyerson, Beth

RESUME AND SUMMARY OF DISCUSSION: This new application entitled “Methadone Patient Access to Collaborative Treatment (MPACT)” is submitted in response to RFA-DA-23-053, HEAL Initiative: Translating Research to Practice to End the Overdose Crisis (R61/R33 Clinical Trial Optional) by Dr. Beth Meyerson from University of Arizona. The application proposes an intervention termed ‘Methadone Patient Access to Collaborative Treatment (MPACT)’ aimed at changing practice in US opioid treatment programs (OTP) to improve methadone maintenance treatment (MMT) retention, decrease in-treatment overdoses and posttraumatic stress symptoms among patients and staff. MPACT includes four evidence-based components that will be pilot tested for their feasibility, effectiveness, and acceptability during the R61 phase in two OTP settings and further refined. During the R33 phase, a randomized control trial will be conducted in 300 clinics with 600 patients and 480 staff to assess the effect of MPACT implementation on culture change in the OTP practices and outcomes for patients and staff.

The application has few strengths. The proposed study is important in addressing the need for evidence-based interventions that target OTP culture and practices and prioritize patient preferences to increase retention in opioid treatment programs. The study’s focus on trauma informed care (TIC) which is relatively understudied is novel. The community based advisory board and involvement of people with lived experience are strengths of the study which is also designed to allow meaningful engagement of the various stakeholders. The use of community based participatory research, CFIR framework and mixed methods approaches further strength the study approach. Participating clinics for the R61 phase have been identified and the team is experienced with the methodology to be employed in the current study. The research environment includes all the resources and infrastructure to support the proposed studies.

The application also has few weaknesses. It is unclear as to how trauma among patients and providers poses significant barriers to treatment retention. Further justification in this regard would strengthen the scientific premise of the study. The application could also use additional details on how the proposed intervention could directly counter the overdose crisis. Several aspects of the aims proposed for the R61 and R33 phases need further clarification including the national level stakeholder engagement planned for the R33 phase. The study could also benefit from considerations of organizational influences and measures to address health inequities or disparities all of which could impact TIC adoption. Although the investigative team has expertise in the relevant study domains, the PI’s rather limited experience with large multisite clinical trials is a concern. The PI’s percent effort during the early stages of the project also seems inadequate considering the significant planning and management efforts required during this time. The overall organization structure of the team could also use further clarifications.

Based on the evaluation of scientific and technical merit, this application received an Overall Impact score of 35.

DESCRIPTION (provided by applicant):
Methadone Patient Access to Collaborative Treatment (MPACT) is a staff-level trauma-informed practice change intervention for US opioid treatment programs (OTP). It is based on the theory that current OTP practice and culture likely contribute to the wide-ranging methadone maintenance treatment (MMT) interruption (>30%) and relapse (>50%) rates known to increase opioid overdoses. OTP treatment culture has been described as “carceral,” and “not healthcare,” with patients reporting being bound to the clinic by required daily supervised medication dosing. Changes in OTP practices have been called for but are recognized as ‘almost impossible.’ US federal regulatory flexibilities intended to facilitate OTP practice change during COVID did not result in wide-spread or sustained MMT delivery changes or service accommodations such as increased multi-day dosing for stable

patients, less frequent urine analyses, or even telehealth. These accommodations would be highly beneficial to rural and home-bound patients. Evidence- based OTP practice change interventions are necessary if the US is going to effectively respond to the opioid overdose crisis. If this lifesaving treatment is available but not well used, we must look to the practice and culture of OTPs. Lack of practice change may be due to staff beliefs and experiences, including their own histories of substance use disorder treatment, traumatic experiences, and on-the-job exposure to vicarious trauma. When staff trauma is addressed, we expect OTP practice orientation will shift from punitive to harm-reduction/patient-centered. MPACT is designed to increase MMT retention and decrease in-treatment overdose and patient- and staff- reported posttraumatic stress symptoms (PTSS). MPACT has four evidence-based components: 1) a 4-module CME/CEU- accredited trauma-informed psychoeducation training program for OTP staff, 2) a trauma navigation model for patients and staff, 3) clinic trauma-informed care (TIC) self-assessment, and 4) separate reflective supervisory structures for counselors/case managers and medical providers. For this proposal, MPACT components will be adapted for OTP settings with patients and providers in a multilevel, trauma-informed planning process with guidance from the Arizona transdisciplinary Drug Policy Research and Advocacy Board comprised of methadone and buprenorphine providers, patients, people with lived/ing drug use experience, harm reduction NGOs, payers, trauma experts, and university researchers. MPACT will be finalized in year 1, pilot tested and refined in year 2, and tested in a cluster-randomized controlled intervention trial in OTP sites across the US in years 3-6. The primary means of gathering data are surveys pushed to staff and patients (baseline and monthly) and retrospective patient chart reviews. We enter a period of unprecedented regulatory change for MMT delivery. MPACT can facilitate and support MMT reform efforts that are planned or already in process. The future of OTPs and methadone treatment depend upon evidence-based OTP practice change interventions. The pivotal question is: Can OTPs adopt Trauma Informed Care (TIC) and, if so, does it improve patient outcomes? MPACT seeks to answer this question in a high risk/high reward proposal. If the answer is YES, then MPACT will be immediately implementable and scalable for any OTP in the US.

PUBLIC HEALTH RELEVANCE

Methadone Patient Access to Collaborative Treatment (MPACT) is a staff-level, trauma-informed practice change intervention for US opioid treatment programs (OTP) to increase treatment retention and decrease in-treatment overdose and patient- and staff-reported posttraumatic stress symptoms (PTSS). It is based on the theory that current OTP practice and culture likely contribute to treatment interruption and relapse rates of 30%-50% which, in turn, increase the risk of opioid overdoses. MPACT will be designed for immediate implementation and scalability; and includes four evidence-based components: 1) a 4-module CME/CEU-accredited trauma-informed psychoeducation training program for OTP staff, 2) a trauma navigation model for patients and staff, 3) clinic trauma-informed care (TIC) self-assessment, and 4) separate reflective supervisory structures for counselors/case managers and medical providers.

CRITIQUE 1

Significance:	5
Investigator(s):	6
Innovation:	2
Approach:	6
Environment:	1

Overall Impact:

This study proposes to implement a comprehensive trauma-informed intervention to support prescribers and patients involved in methadone-dispensing opioid treatment programs. This Type 1 trial will assess the effectiveness of the proposed intervention on overdose in addition to implementation outcomes. The study is focused on workforce development and training. Strengths include the

emphasis on trauma in the substance use treatment workforce and the need for substantial practice changes in opioid treatment programs. Substantial weaknesses, however, limit the potential impact of this study. Weaknesses include the principal investigator who has minimal experience leading large, multi-site trials, inconsistency regarding the primary outcomes, and inadequate preliminary data demonstrating that vicarious trauma is a barrier to practice change and the successful delivery of methadone treatment.

1. Significance:

Strengths

- Increasing access to medications for opioid use disorder is tremendously important and current ASAM guidelines support the need for access to all evidence-based medications to provide flexibility for individual patient needs.
- Trauma is critically understudied in substance use service provision and assessing the impact of provider and patient trauma on methadone retention is important.

Weaknesses

- A significant weakness is the lack of clear data on whether addressing trauma among providers and patients will change retention outcomes. It seems more likely that regulatory changes will have greater impact which is why there have been renewed discussions about de-regulating methadone. Although the researchers state limited evidence during the public health emergency to suggest the effectiveness of de-regulation, this is an area of continued study and of significance to public health. It is a big leap to state that de-regulation has proven ineffective and additional evidence is needed to suggest that provider-level trauma is a significant barrier to treatment retention.

2. Investigator(s):

Strengths

- The research team includes multiple co-investigators with significant NIH funding and biostatistical expertise.

Weaknesses

- The PI does not appear to have enough time dedicated in the early years of the project given the significant project management associated with developing and implementing the proposed trial.
- The PI has limited experience leading large clinical trials. Dr. Crosby will serve as a mentor, but it is unclear whether this time commitment is sufficient to support the proposed study.

3. Innovation:

Strengths

- The CBPAR approach is important for intervention development and testing. The investigators have fostered an innovative advisory board to serve as a community partner.
- The study includes plans to develop a pipeline of NIDA researchers with lived experience which is critically important to the field.

Weaknesses

- None noted

4. Approach:

Strengths

- The study team has conducted several preliminary studies documenting barriers to OTP practice changes and has experience using many of the methods proposed for this study, including previously validated instruments.
- The study will generate useful data on MPACT feasibility, acceptability, and appropriateness.

Weaknesses

- The preliminary studies do not provide adequate support that trauma is an important barrier to OTP practice change.
- Why Type 1? MPACT could be conceptualized as an implementation strategy to support use of methadone, a known evidence-based practice. If so, a Type 3 might be a more appropriate design to see if addressing trauma supports successful methadone treatment delivery while still measuring important clinical outcomes such as overdose. I think the proposal would be more compelling with implementation outcomes as primary, including provider willingness to make recommended changes to OTP practice, and the ability to track overdose data more accurately.
- What steps will the team take if there is a low survey response rate from OTPs in Aim 3?
- The national recruitment strategy for the R61 trial is ambitious. The study team should better demonstrate their ability to recruit diverse OTPs into the research study and include some letters of support beyond the existing clinic partners.

5. Environment:

Strengths

- There is an excellent network developed with strong partnerships in Arizona, a community-based advisory board already in place.
- The University of Arizona, Indiana University, and the University of Kentucky all have excellent research infrastructure to support the study activities.

Weaknesses

- None noted

CRITIQUE 2

Significance:	4
Investigator(s):	2
Innovation:	4
Approach:	3
Environment:	2

Overall Impact:

This application plans a staff-level trauma informed practice change intervention to address the high rates of treatment interruption and chronic drug use relapse for methadone maintenance treatment (MMT) clients. If successful, this intervention will provide a good model for agencies to promote culture change toward client centered approaches and better treatment outcomes. Additionally, this application has the potential to better understand the prevalence of vicarious trauma and PTSS among MMT staff and promote workforce health by identifying and treating these conditions. The investigative team has plans to facilitate a national survey of MMT sites to better understand what client centered practices

they are currently utilizing and collect their attitudes about the intervention. This will be fruitful information to help with developing an intervention that is broadly applicable and generalizable beyond the clinical trial sites. While these are some strengths, there are also a few weaknesses. For instance, how is the primary outcome measure of treatment interruption defined? Treatment can be interrupted in part by hospitalization (sometimes not related to drug use) or incarceration; how much will the intervention reasonably impact these outcomes? Additionally, it's unclear from this application whether there is previous research to support the concept that TIC training or improved TIC attitudes relates to client centered approaches or to better client outcomes (such as treatment retention). An example from another/related field would be helpful. Additionally, consideration of adding a baseline period to compare to the intervention and control condition; and additional details on how the R61 aim 1 will be implemented.

1. Significance:

Strengths

- This study proposes to address the high rates of treatment interruption and chronic drug use relapse for MMT clients, related to overdose, by promoting a trauma informed MMT patient centered approach within these organizations. The approach also addresses vicarious trauma and PTSS among staff. The investigators sight instances where lack of attention to trauma histories have shown to result in lack of patient empathy. This project will provide a better understanding of the prevalence of vicarious trauma and PTSS among MMT staff, and how these conditions relate to patient centered care practices.
- Additionally, the investigator team points out many instances where patient centered care references are trumped by organizational convenience, the protocol will specifically prioritize client preferences in care delivery.

Weaknesses

- It's unclear from this application whether there is previous research to support the concept that TIC training or improved TIC attitudes relates toward client centered approaches or to better client outcomes (such as treatment retention). An example from another/related field would be helpful.
- The application describes a training model that they propose will improve client outcomes such as treatment retention and PTSS outcomes within OUD settings; while these are important and connected with overdose, it's unclear whether the study is squarely relevant to addressing the overdose crisis (the specific purpose of this funding announcement).

2. Investigator(s):

Strengths

- The investigator team have experience with harm reduction, MOUD, trauma, opioid overdose prevention, and statistics and analysis. They have conducted studies/produced publications that have advanced the field.
- Intention to place a postdoc with lived experience.

Weaknesses

- None noted

3. Innovation:

Strengths

- Integrating trauma informed practices into MMT clients is understudied.
- This project has the potential to inform how incorporating TIC practices in MMT could advance openness to culture change around client centered care practices. If successful, this approach would help other fields of addiction medicine and practice.

Weaknesses

- On the surface, this application does not offer innovative approaches that would advance methodology or instrumentation.

4. Approach:

Strengths

- The R61 approach includes co-creation of the project by both patient-provider stakeholders, a pilot phase with 2 sites to pilot the intervention, and a randomized national sample of MMT sites surveyed on the feasibility of the study (that include a data completeness check). This approach will set-up the necessary conditions for the subsequent R33 phase. The stakeholder group is already established. The 4 step intervention addresses psychoeducation on TIC for staff, a model for gaining access to trauma informed care for staff and clients in need, assessment of change in TIC organizational practices, and supervisory modeling of TIC for staff.
- The measures are broadly utilized in prior TIC studies and are appropriate for this study design.
- The R33 phase has a strong stratification approach including randomizing sites to condition based on state regulatory favorability toward OTPs telehealth and clinic visits. Additionally, the site recruitment approach is well planned with some flexibility anticipated based on R61 results.
- The project will use a hybrid type 1 clinical trial with the intervention (trauma informed care) as the evidence-based practice, and with exploration of how inner and outer factors impact adoption of the intervention. This seems appropriate as TIC has minimally been used within MMT settings as a change mechanism.

Weaknesses

- The primary outcome measure is retention, defined as time to treatment interruption. First, how is treatment interruption defined? If it includes hospitalization, incarceration; how much will the intervention reasonably impact these outcomes?
- Regarding the R61 approach, the application lacks enough detail on how aim 1 will be conducted, specifically, how will staff and clients get access to materials for review? The protocol states that these participants will complete anonymous surveys to provide suggestions and feedback. While that is great for some folks, it’d also be good to offer group discussion for consensus building, perhaps in focus groups specific to role (client vs. staff sessions).
- Regarding the use of client focused practices, it’s possible that while staff express efficacy and support in using the approaches, beyond state regulatory favorability toward OTPs, the organizational leadership and structure may not allow their use. Measures and intervention approaches to better understand and address organizational level influences on TIC adoption would be useful.
- During the R33 phase, the plan that includes expanding to a national level stakeholder model could be a bit ambitious.
- The application does not address how health inequities or disparities in relevance to the intervention will be addressed or studied.

- If addressing vicarious trauma and PTSS among MMT staff is one of the intervention pillars; then perhaps consider measuring job satisfaction, burnout, and job retention prior to and after receiving 'trauma navigation'.
- Will there be a baseline period where the outcome rates will be compared to rates during the intervention versus the control arm?

5. Environment:

Strengths

- The environment seems adequate for this study, through collaboration with UKentucky, Indiana, and primary center at University of Arizona.

Weaknesses

- None noted

CRITIQUE 3

Significance:	1
Investigator(s):	1
Innovation:	1
Approach:	3
Environment:	1

Overall Impact:

This proposal aims to investigate Methadone Patient Access to Collaborative Treatment (MPACT) a staff-level, trauma-informed practice change intervention for US opioid treatment programs (OTP) to increase treatment retention and decrease in-treatment overdose and patient- and staff-reported posttraumatic stress symptoms (PTSS). The proposed study team has significant expertise in relevant areas (e.g., substance use, implementation science, CBPR), and has a history of collaboration; the study team is enriched through the meaningful incorporation of a Drug Policy and Advocacy Board (reflecting individuals with expertise via lived experience) that has been working with the study team since 2020. Strengths of the application include: 1) developing an intervention aimed at improving the culture of OTP settings using a participant-driven and supported approach; 2) sites have already been recruited for the pilot phase of the trial (supported by LOSs); 3) innovative intervention approach that leverages existing partnerships and appropriate methodology for studying the intervention implementation and preliminary impact.

1. Significance:

Strengths

- Addresses OTP practices and culture as a mechanism through which MMT retention may be impacted (via MMT delivery).
- Leverages an existing advisory board (DPAB) that has been working with the study team since 2020.
- Findings from this study have the potential to be immediately scalable and implementable in OTOP settings nationally, given the utilization of in-place structures, existing tools and methods.

Weakness

- None noted

2. Investigator(s):

Strengths

- This is an PI-led study by Dr. Beth Meyerson at the University of Arizona. Dr. Meyerson has extensive expertise working with substance using populations, healthcare settings working with these individuals, and other related systems partners (e.g., policy makers).
- Also leverage the expertise of stakeholder groups (e.g., DPRAB) facilitated via the PI's CBPR approaches.
- The investigative team has an established history of productive collaboration including work with the target population and community partners for this grant positioning them well to be able to initiate work quickly and meet the proposed study goals within the timeframe.

Weaknesses

- The PI / Co-I structure was a bit confusing to understand in some sections. It was clear that overall leadership will be provided by Dr. Beth Meyerson, however, additional detail would have been useful.

3. Innovation:

Strengths

- Utilizes a patient and provider driven change-model, with meaningful involvement from these stakeholder groups to influence MMT practice change to support patient engagement and health outcomes.
- The intervention is innovative in its approach and setting (i.e., incorporating TIS practices into OTOP setting.

Weaknesses

- None noted

4. Approach:

Strengths

- Thoughtful approach to incorporating individuals with lived experience and the population of interest and leveraging the CBPR principles; separating patient and providers into different planning groups is responsive to embedded power structures. Also, expansion of the state-level DPRAB to the national-level.
- Utilization of CFIR throughout the proposed study; the study team has previous experience applying this IS approach in their work with this population.
- Mixed methods data collection and data collected from multiple sources and levels (patients, providers, systems) in the R61 phase.
- The study team has already identified clinics to participate in R61 phase.
- Hybrid Type 1 cluster RCT design in R33 phase.
- Clear description of processes to finalize trial procures in R33 phase (e.g., measures, frequency of data collection).
- The PI has employed methods proposed in the R33 phase in previous studies in order to recruit clinics for participation successfully.
- Data review and iterative feedback to community partners is well-thought out and described.

- Has the potential to strengthen the pipeline of NIDA-funded researchers with lived/living experience in substance use and SU treatment

Weaknesses

- The pilot phase is short, and design is ambitious- however- the applicants have established partnerships and sites for this phase.
- The applicants attempt but don't explicitly focus on overdose (as is noted in the RFA) due to challenges with measurement in OTP settings. The applicants should discuss the limitations in this context.
- The R33 is very complex and reference that one study coordinator will complete clinic-level fidelity checks without provided detail about these processes to ensure that they are feasible with this level of effort.
- The application does not address how health inequities or disparities in relevance to the intervention will be addressed or studied.

5. Environment:

Strengths

- Supportive environment at the University of Arizona, and the University of Kentucky and the subrecipients.
- Strong LOS from OTP partner in the R61 phase: Community Medical Services

Weaknesses

- None noted

THE FOLLOWING RESUME SECTIONS WERE PREPARED BY THE SCIENTIFIC REVIEW OFFICER TO SUMMARIZE THE OUTCOME OF DISCUSSIONS OF THE REVIEW COMMITTEE ON THE FOLLOWING ISSUES:

STUDY TIMELINE: ACCEPTABLE

PROTECTION OF HUMAN SUBJECTS: ACCEPTABLE

INCLUSION OF WOMEN PLAN: ACCEPTABLE

INCLUSION OF MINORITIES PLAN: ACCEPTABLE

INCLUSION OF INDIVIDUALS ACROSS THE LIFESPAN: ACCEPTABLE

VERTEBRATE ANIMALS: NOT APPLICABLE

BIOHAZARD COMMENT: NOT APPLICABLE

FOREIGN INSTITUTION: NOT APPLICABLE

SELECT AGENTS: NOT APPLICABLE

RESOURCE SHARING PLAN: NOT APPLICABLE

AUTHENTICATION OF KEY BIOLOGICAL AND/OR CHEMICAL RESOURCES: NOT APPLICABLE

COMMITTEE BUDGET RECOMMENDATIONS: The Budget is recommended as requested.

ADMINISTRATIVE NOTE FROM SRO: The font used for the 'Milestones and Timeline' table is too small and illegible. Please note that while smaller font size is permissible in figures/charts/diagrams, it must be legible when the document is viewed at 100%. Please see link below for further details.

https://grants.nih.gov/grants/how-to-apply-application-guide/format-and-write/format-attachments.htm

---

Footnotes for 1R61DA059889-01; PI Name: Meyerson, Beth

NIH has modified its policy regarding the receipt of resubmissions (amended applications).See Guide Notice NOT-OD-18-197 at https://grants.nih.gov/grants/guide/notice-files/NOT-OD-18-197.html. The impact/priority score is calculated after discussion of an application by averaging the overall scores (1-9) given by all voting reviewers on the committee and multiplying by 10. The criterion scores are submitted prior to the meeting by the individual reviewers assigned to an application, and are not discussed specifically at the review meeting or calculated into the overall impact score. Some applications also receive a percentile ranking. For details on the review process, see http://grants.nih.gov/grants/peer_review_process.htm#scoring.

MEETING ROSTER

The roster for this review meeting is displayed as an aggregated roster that includes reviewers from multiple DA Special Emphasis Panels Meetings
for the 2023/10 council round.
This roster for DA is available here.
